# Medical imaging and physiological modelling: linking physics and biology

**DOI:** 10.1186/1475-925X-8-1

**Published:** 2009-01-12

**Authors:** Manuchehr Soleimani, Rebecca J Shipley, Nic Smith, Cathryn N Mitchell

**Affiliations:** 1 Department of Electronic and Electrical Engineering, University of Bath, Bath, UK; 2 Mathematical Institute, University of Oxford, Oxford, UK; 3 Computing Laboratory, University of Oxford, Oxford, UK

Medical image analysis is increasingly providing a sophisticated set of tools for processing measurement inputs into clinically relevant outputs, although this is, on the whole, completed without consideration of the underlying physiology. In contrast, physiological modelling provides a predictive tool based on a physical and biological understanding of the underlying processes. In this editorial, we discuss the possibility of integrating physiological modelling data with medical images and measurements with the goal of providing new types of physiologically meaningful information with increased clinical relevance.

Underpinning this goal are advances in conventional imaging methods such as MRI and PET and their multimodal combinations. These provide the ability to acquire high quality images for characterising a wide range of physiological functions. A drawback to the use of these technologies is the high purchase and running costs. However emerging imaging techniques, such as ultrasound and electromagnetic imaging [1] are now beginning to provide compelling cost effective alternatives in a range of contexts.

In this editorial, we focus on the benefit of this combined approach in four key areas: image and model validation, multimodal imaging, regularization processes and model customization. Finally, we present two examples that serve to demonstrate how the potential of a combined imaging/modelling approach is already being realised.

An existing interaction between medical imaging and modelling is mutual validation – the process of comparing data from model and imaging systems. This integration has numerous advantages from both modelling and imaging perspectives. Firstly, validating model predictions against imaging data provides a mechanism for testing that the model captures all the key physiological components of the system. This is performed for a prescribed set of parameter values, and once complete the model is a powerful tool to establish predictions of the system properties in new regimes. From an imaging perspective, models can be used to extract information that is not directly available from the images themselves and thus aid clinical diagnosis. For example, the mechanical stress or work of a contractile tissue can not be detected directly from an image but is straight forward to extract from a model parameterised from same information. This 'in silco imaging' approach provides significant capacity to define new metrics for focusing clinical trials, optimising patient selection and customising therapy.

An extension of this validation process is the integration of model simulation with multiple imaging protocols. Combined with registration techniques, this multimodal approach is increasingly providing complementary data within a common reference frame. This in turn assists the rationalisation of conflicting clinical metrics and the resulting different diagnosis. In silco imaging is perhaps even more relevant in the multimodality context where biophysically based models (e.g. electrical, solid or fluid mechanical) can provide yet further information: one successful example is in the detection and monitoring of breast cancer through the use of magnetic resonance (MR) imaging and mammography. Registration of the two sets of images is fundamental to the success of the process; however, the patient's posture differs under each of the modalities, and the breast is compressed during mammography so co-registration is extremely difficult to achieve. In [2] the authors develop a 3D, patient-specific, elasticity-based model that describes the deformation of the breast under mammography. Further, this model can be used as a tool for co-registration between the sets of images acquired through the different imaging modalities. This serves as an excellent example of how a mechanical model can provide independent, yet meaningful, information to inform the imaging process.

However, various challenges remain in the co-registration between images acquired from multiple modalities and the use of models to interpret the results. In particular, accurate parameter extraction is a significant problem because the image reconstruction process is often an ill-posed inverse problem. Regularization techniques are used to address the associated lack of uniqueness and stability in the reconstruction process [1], however the majority of these techniques are based on a simplified, non-physical mathematical expression such as smoothness of the imaging area. Substituting a physiological model has significant potential to provide a mechanism for a physically motivated regularization process, and indeed some preliminary studies have shown significant improvement in image quality in this way (see for example [3]). The physiological models can provide meaningful and independent spatial and temporal regularization.

To successfully implement this combined approach, it is essential that the mathematical models are sophisticated enough to capture the key physiological features of the system. This is a mathematical challenge in its own right; tissues have anisotropic and multi-scale properties that must be realised in mathematical models and solved on the complex geometries. In addition, the material and physiological properties of the setup appear as parameters in the mathematical models, and in this way the physiological models must be customized through inputting patient-specific structural and functional information. Some of this information can be obtained directly from currently available modalities. This includes geometry, motion and to more limited extents perfusion, biochemical markers and electrical conduction. However, in general the necessary parameters are frequently difficult to measure in vivo and so realistic parameter estimation can be a real limitation to the application of these models. An interdisciplinary approach encourages correlation between model parameters and image quantities that may resolve some of these challenges. Considerable progress in these arenas has recently been made, most notably, under the banners of international collaborative efforts, such as the Physiome and Virtual Physiological Human projects [4]. Within these initiatives, the need to overcome bottlenecks in model development has motivated the successful development of software platforms and mark-up languages to extract parameters across multiple spatial scales, simulate behaviour and visualize results. To tangibly illustrate some of the potential and progress of the approach we consider two key examples of integrated biophysically based models.

Arguably one of the more advanced examples of an integrated set of physiological models that collectively focus on a single organ system is the cardiac modelling literature [5]. At the cellular level electro-mechanical models now include detailed representations of membrane-bound channels, transporter functions and protein interactions. These frameworks have provided a successful paradigm for integrating individual data sets, and for interpreting the ensemble behaviour of both electrical and mechanical recordings in a range of normal and patho-physiological conditions. High performance computing and multi-scale modelling techniques have more recently enabled the embedding of these cellular models in spatial distributed tissue domains. Central to representing structure within these models is the integration of data source from confocal, CT and MR (among other modalities) that reveal the myocyte, fibroblast, and collagen microstructure. This has been successfully used to determine the conductivity and stiffness tensor within these continuum models to predict the functional properties of electrical conductivity and mechanical stiffness of the whole heart [4]. The next step for the cardiac field is to parameterise and customise these models for humans using only minimally-invasive clinical measurements. As outlined above successful application of parameter identification and validation techniques for model customisation is a difficult mathematical and computational problem. However, developing robust solutions will be crucial for enabling patient-specific simulations.

The second example is the modelling of tumour treatment by anticancer agents that requires insight into the mechanisms by which the drug is delivered to the cancerous tissue. For therapeutic drugs administered intravenously, this necessitates an understanding of the flow of blood through the tumour vasculature and surrounding porous interstitium of the tumour, as well as knowledge of the drug transport characteristics. Multi-scale models of the tumour vasculature have been developed [6]; however, to maximize their potential these must be combined with, and validated against, vasculature imaging data. In this way, it is possible to develop full models of drug delivery based on the vascular properties of the tumour, and so predict drug penetration into the interstitium, together with the extent of cell kill and tumour regression.

It is also important to mention the role of statistical techniques both in physiological modelling and multi-modal imaging. An example of complex system is human brain. In [7] a combination of high temporal contrast monitoring techniques (e.g. EEG and MEG) and high spatial resolution imaging technique (e.g. fMRI) used to understand the system. The association of imaging parameters with genetic variations of neurotransmitter systems allows the investigation of neurotransmitter activity. To overcome the limitations of standard statistical methods, new approaches in machine learning have been adapted to handle multiple parameters obtained from brain imaging, modelling and genetic measurements.

In conclusion, the combination of modelling and imaging has the potential to open a number of new opportunities. The long-term aim is to embrace the power of the modelling tools being developed and integrate them with clinically, scientifically and economically effective imaging techniques to achieve the goal of universal healthcare. It is important noticing that a combined physiological modelling and medical imaging can play role in various aspects of healthcare system including screening and treatment. Before they can be used, there is a need for validation of the combined techniques. By very nature of human physiology it is likely that this validation requires an international level collaboration, especially by providing data and establishing various benchmarks. The combination of modelling and imaging together could help realise automatic diagnosis and early recognition of heath problems from routine health checks. Further, there is the potential for predicting the outcome of a given treatment plan through the formation of patient specific models. The realisation of this requires extensive interdisciplinary research, encompassing mathematicians, biologists, engineers and most importantly clinicians. The research community could contribute to this by organizing themed conferences that bring experts from diverse disciplines together to develop a common view of the optimal way forward. In this way, collaborations will be developed that capitalise on internationally-leading expertise in the necessary research themes.

## References

[B1] Soleimani M (2006). Electrical impedance tomography imaging using a priori ultrasound data. Biomedical Engineering Online.

[B2] Pathmanathan P, Gavaghan D, Whiteley J, Brady M, Nash M, Nielsen P, Rajagopal V (2004). Predicting tumour location by simulating large deformations of the breast using a 3D finite element model and Nonlinear Elasticity. Lecture Notes in Computer Science.

[B3] Poplack SP, Paulsen KD, Hartov A, Meaney PM, Pogue BW, Tosteson TD, Grove MR, Soho SK, Wells WA (2004). Electromagnetic breast imaging: Average tissue property values in women with negative clinical findings. Radiology.

[B4] Crampin EJ, Halstead M, Hunter PJ, Nielsen PF, Noble D, Smith NP, Tawhai MH (2004). Computational physiology and the Physiome project. Experimental Physiology.

[B5] Smith NP, Crampin EJ, Niederer SA, Bassingthwaighte J, Beard DA (2007). Computational Biology of Cardiac Myocytes: Proposed Standards for the Physiome. Journal of Experimental Biology.

[B6] Chapman SJ, Shipley RJ, Jawad R (2008). Multiscale modelling of fluid transport in vascular tumours. Bulletin of Mathematical Biology.

[B7] Gallinat J, Heinz A (2006). Combination of multimodal imaging and molecular genetic information to investigate complex psychiatric disorders. Pharmacopsychiatry.

